# Supplementary figures and images for: Trend of liver cancer attributable to alcohol use in China from 1992 to 2021: An age-period-cohort analysis study
Source: PLoS One. 2026 Mar 18;21(3):e0343137. doi: 10.1371/journal.pone.0343137 (PMC12998816; doi:10.1371/journal.pone.0343137)

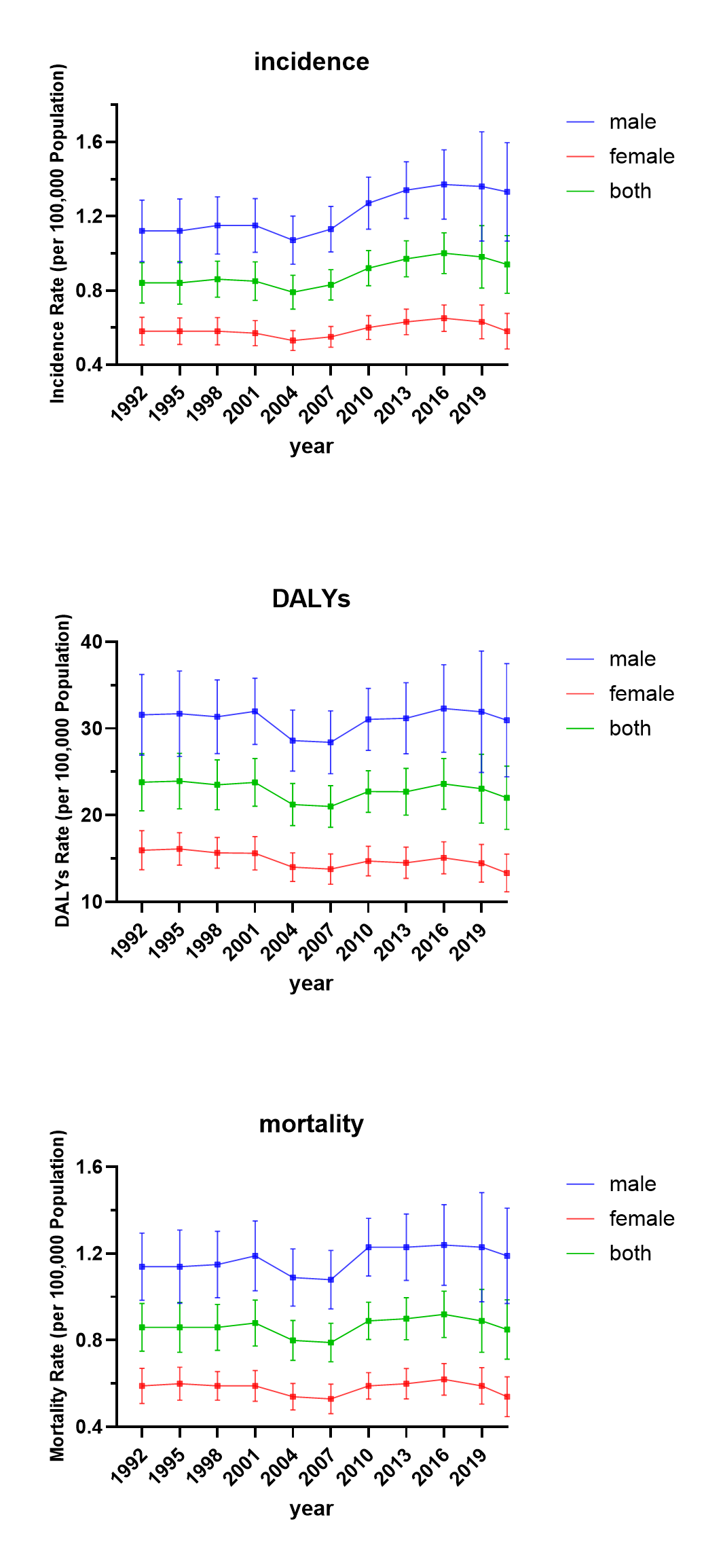

Supplement: S1 Fig — (TIF) [file pone.0343137.s001.tif]

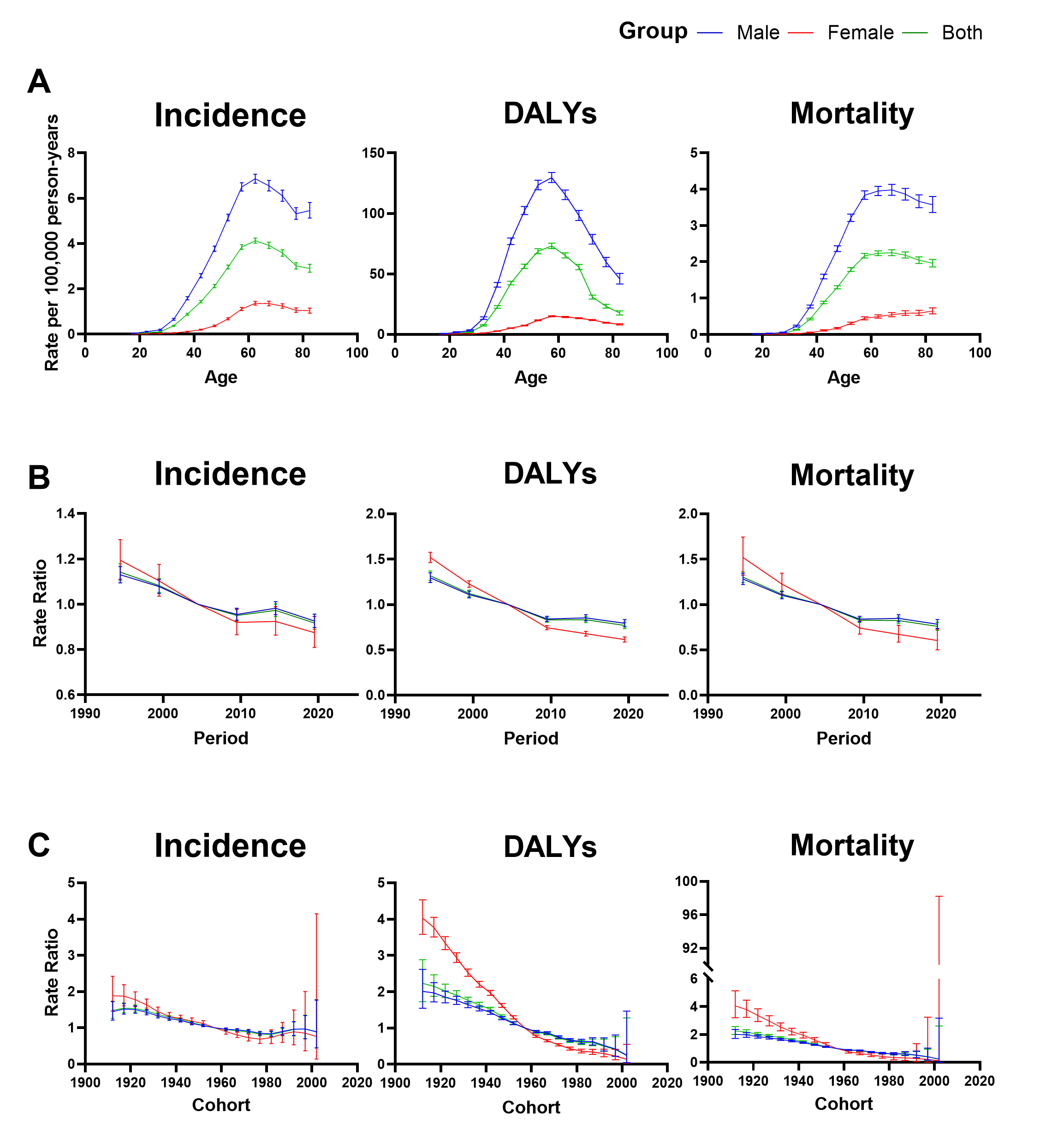

Supplement: S2 Fig — (TIF) [file pone.0343137.s002.tif]
