# Supplementary material for: Trend of liver cancer attributable to alcohol use in China from 1992 to 2021: An age-period-cohort analysis study
Source: PLoS One. 2026 Mar 18;21(3):e0343137. doi: 10.1371/journal.pone.0343137 (PMC12998816; doi:10.1371/journal.pone.0343137)
Supplement: Attachment — Submitted filename: Response to Reviewers.docx [file pone.0343137.s004.docx]

Dear Editor，

We sincerely thank you for your thoughtful and constructive comments on our manuscript. These insights have been invaluable in helping us improve the quality and clarity of our work. We have carefully addressed all the points raised, and a point-by-point response is provided below. All changes in the manuscript have been highlighted in red for your convenience.

**Reviewer #1:**

1) **There is a lack of detail on how data quality was assessed. Explicit information on how GBD data for china were validated should be added to the manuscript.**

**Response:**We thank the reviewer for this important suggestion. The GBD study complies with the Guidelines for Accurate and Transparent Health Estimation Reporting. The prevalence and incidence data of Liver Cancer Attributable to Alcohol Use in China in the GBD database are mainly derived from national authoritative databases (e.g., the National Center for Disease Control and Prevention, the National Cancer Center and the National Bureau of Statistics of China). Currently, there have been tens of thousands of studies worldwide that have used the GBD database for analysis, and many of them are from China. These studies have been used to guide China's public health policies, thus verifying the validity and reliability of the database. We have now added a detailed description of the GBD data for China in the "Data collection" subsection within the "Methods"section.

2) **Were multiple comparisons controlled for in the analyse? Is there the potential for inflated Type-I error? Either this statistical control should be used, or an explanation on why it is not needed should be provided.**

**Response:**We appreciate the reviewer's keen methodological point. The Joinpoint software currently uses a series of permutation tests to determine the number of connection points. Before version 3.0, the software used Bonferroni adjustments to control the error probability of each multiitem test. The Bonferroni adjustment is relatively conservative because the actual overall significance level is usually lower than the nominal level α. The permutation test controls the overall probability of overfitting error and is superior to the traditional Bonferroni adjustment. Therefore, the permutation test used in this study significantly controls the probability of making type 1 errors.We have now explicitly addressed this in the "Statistical Analysis" section.

3) **The project determined that the incidence of DALYs declines after age 65. However, could this reflect mortality levels (i.e., individuals with liver disease died at earlier ages) rather than a true reduction in DALYs? In this case, I believe this data would be censored and may need to be accounted for. This should be discussed as a potential limitation or controlled for.**

**Response:**We fully acknowledge that the interpretation of the declining DALYs trend in the elderly population (≥65 years) may be influenced by this bias. Specifically, individuals who died from alcohol-attributable liver cancer before reaching the older age groups are not represented in the DALYs calculations for those groups, potentially leading to an underestimation of the true burden in the elderly and an overestimation of the magnitude of the decline. As correctly noted by the reviewer, and due to the nature of our secondary data analysis, we are unable to directly observe or statistically adjust for this effect at the individual level within the current GBD framework. Therefore, we have taken great care to discuss this limitation transparently in the revised manuscript (please see the 'Discussion'' sections), framing our conclusions with appropriate caution. We note that advanced statistical methods such as the Fine-Gray competing risk model or multi-state modeling with individual-level time-to-event data, could potentially help quantify and adjust for this bias. Addressing this complex issue with more granular data constitutes a valuable and clear direction for our future research.

4) **The figures are difficult to see as they are very blurry. Please provide more appropriate figures for interpretation.**

**a. Further, it appears that no confidence intervals are included on Figure 1. It is difficulty to see.**

**Response:**We sincerely apologize for the suboptimal quality of the figures in our initial submission.The new figure now displays the raw trends for incidence, DALYs, and mortality. Data points at regular intervals (every 3-5 years) have been marked and labeled with their specific values for males, females, and both sexes combined.

**Reviewer #2:**

**To my opinion, the manuscript is well-written and demonstrates the interesting results for researchers working in similar area as authors. But still I have some recommendations how to improve the quality of the manuscript. To my opinion results presented in Figure 1 partially duplicates the results from Tables 1-3. So I recommend remove from the Figure 1 results of Joinpoint analysis which are already presented in the mentioned Tables. Instead of Joinpoint results in the Figure 1, I recommend indicate the specific values of the incidence, mortality, and DALYs let say each three years of the period between 1992 and 2021 on the lines separately for male, female and all population. You also need indicate in what values are presented in the parts A,B, and C of the Figure one.**

**Response:**We thank the reviewer for the positive feedback and the excellent suggestion to improve the clarity and information value of Figure 1.

We have revised Figure 1 accordingly. The joinpoint segments have been removed. The new figure now displays the raw trends for incidence, DALYs, and mortality. Data points at regular intervals (every 3-5 years) have been marked and labeled with their specific values for males, females, and both sexes combined. The y-axis labels now clearly state that rates are presented as "per 100,000 population".

**Journal Requirements:**

We have thoroughly reviewed the PLOS ONE formatting guidelines (including manuscript structure, headings, and file naming) and ensured full compliance. All figure files have been renamed according to the journal’s requirements (e.g., “Fig1.tif”), and the manuscript layout (such as heading levels, citation formats, and equation formatting) has been adjusted to meet the specified standards.

**The accurate grant numbers are confirmed as follows:**

National Science and Technology Major Project(2024ZD0523904)

Science and Technology Department of sichuan Province(2025JDKP0057)

The funders had no role in study design, data collection and analysis, decision to publish, or preparation of the manuscript.

We have removed all funding-related text from the manuscript.

We have added separate, comprehensive captions for each figure in the manuscript. Each caption includes the figure title.

We sincerely appreciate the editors’ and reviewers’ valuable time and constructive comments, which have helped improve the quality of our manuscript. We hope the revised version meets the journal’s publication standards and look forward to your further feedback.

Sincerely,

Dan Jia

E-mail : 417522774@qq.com
